# Monitoring Health Care Workers at Risk for COVID-19 Using Wearable Sensors and Smartphone Technology: Protocol for an Observational mHealth Study

**DOI:** 10.2196/29562

**Published:** 2021-05-12

**Authors:** Caroline A Clingan, Manasa Dittakavi, Michelle Rozwadowski, Kristen N Gilley, Christine R Cislo, Jenny Barabas, Erin Sandford, Mary Olesnavich, Christopher Flora, Jonathan Tyler, Caleb Mayer, Emily Stoneman, Thomas Braun, Daniel B Forger, Muneesh Tewari, Sung Won Choi

**Affiliations:** 1 Division of Pediatric Hematology/Oncology Department of Pediatrics University of Michigan Ann Arbor, MI United States; 2 Division of Hematology and Oncology Department of Internal Medicine University of Michigan Ann Arbor, MI United States; 3 Department of Mathematics College of Literature, Arts, and Sciences University of Michigan Ann Arbor, MI United States; 4 Department of Internal Medicine Division of Infectious Diseases University of Michigan Ann Arbor, MI United States; 5 Department of Biostatistics School of Public Health University of Michigan Ann Arbor, MI United States; 6 Rogel Cancer Center University of Michigan Ann Arbor, MI United States; 7 Department of Computational Medicine and Bioinformatics University of Michigan Ann Arbor, MI United States; 8 Department of Biomedical Engineering University of Michigan Ann Arbor, MI United States

**Keywords:** mobile health, app, mHealth, wearable, sensor, COVID-19, health care worker, frontline worker, smartphone, digital health

## Abstract

**Background:**

Health care workers (HCWs) have been working on the front lines of the COVID-19 pandemic with high risks of viral exposure, infection, and transmission. Standard COVID-19 testing is insufficient to protect HCWs from these risks and prevent the spread of disease. Continuous monitoring of physiological data with wearable sensors, self-monitoring of symptoms, and asymptomatic COVID-19 testing may aid in the early detection of COVID-19 in HCWs and may help reduce further transmission among HCWs, patients, and families.

**Objective:**

By using wearable sensors, smartphone-based symptom logging, and biospecimens, this project aims to assist HCWs in self-monitoring COVID-19.

**Methods:**

We conducted a prospective, longitudinal study of HCWs at a single institution. The study duration was 1 year, wherein participants were instructed on the continuous use of two wearable sensors (Fitbit Charge 3 smartwatch and TempTraq temperature patches) for up to 30 days. Participants consented to provide biospecimens (ie, nasal swabs, saliva swabs, and blood) for up to 1 year from study entry. Using a smartphone app called Roadmap 2.0, participants entered a daily mood score, submitted daily COVID-19 symptoms, and completed demographic and health-related quality of life surveys at study entry and 30 days later. Semistructured qualitative interviews were also conducted at the end of the 30-day period, following completion of daily mood and symptoms reporting as well as continuous wearable sensor use.

**Results:**

A total of 226 HCWs were enrolled between April 28 and December 7, 2020. The last participant completed the 30-day study procedures on January 16, 2021. Data collection will continue through January 2023, and data analyses are ongoing.

**Conclusions:**

Using wearable sensors, smartphone-based symptom logging and survey completion, and biospecimen collections, this study will potentially provide data on the prevalence of COVID-19 infection among HCWs at a single institution. The study will also assess the feasibility of leveraging wearable sensors and self-monitoring of symptoms in an HCW population.

**Trial Registration:**

ClinicalTrials.gov NCT04756869; https://clinicaltrials.gov/ct2/show/NCT04756869

**International Registered Report Identifier (IRRID):**

DERR1-10.2196/29562

## Introduction

### Background

A novel coronavirus (SARS-CoV-2) causing severe respiratory illness (COVID-19) was first reported in a cluster of individuals in the city of Wuhan, Hubei Province, China on December 31, 2019 [1]. The cases quickly spread beyond Wuhan to other parts of China and to many other countries. In the United States, the first case of COVID-19 was confirmed in Washington State on January 19, 2020 [2]. By January 31, 2020, the World Health Organization declared the outbreak a Public Health Emergency of International Concern and a global pandemic by March 11, 2020. As of March 23, 2021, there were nearly 124.5 million confirmed cases worldwide and over 2.7 million deaths. More than 30.5 million cases and 556,000 deaths occurred in the United States alone [3].

COVID-19 is spread by human-to-human transmission via droplets or direct contact. The symptoms of COVID-19 infection appear after an incubation period of 5.5 days on average [4]. The most common symptoms at the onset of COVID-19 illness include fever, cough, and fatigue. In addition, muscle or body aches, headache, new loss of taste or smell, sore throat, and congestion have been commonly reported [5]. During the early months of the pandemic, much of the world’s population faced government-mandated lockdowns to mitigate transmission. Social and behavioral science insights were being widely deployed in an effort to improve public health [6]. The most effective strategies to mitigate the spread of this novel viral respiratory illness were nonpharmaceutical—social distancing, quarantine, and isolation [7]. Indeed, data from the severe acute respiratory syndrome–related coronavirus outbreak in 2002 suggested that the psychosocial burden of these health measures including quarantine was wide-ranging, substantial, and long-lasting [8]. However, the magnitude of the COVID-19 pandemic was unlike any other in our lifetime, so the impact that these widespread lockdown strategies would have on health-related quality of life (HRQOL) was largely unknown [9].

### Prior Work

Health care workers (HCWs) are at increased risk for many infections including COVID-19. A large-scale observational study from early in the pandemic found that HCWs were over 11 times more likely to report a positive COVID-19 test than the general population and still over three times more likely when that statistic was adjusted for increased testing frequency among HCWs [10]. Numerous studies have reported nosocomial outbreaks of COVID-19 affecting both patients and HCWs at hospitals and long-term care facilities throughout the United States [11-13]. Fortunately, health care facilities have implemented universal masking policies and increased their testing capacity as the pandemic has progressed; these practices have mitigated some of the risks of transmission to HCWs [14,15]. Despite these improvements, the efficacy of safety precautions is limited by compliance and availability of supplies, leaving HCWs at risk. Thus, monitoring HCWs for COVID-19 is critical for protecting HCWs themselves and for protecting their patients, their families, and the health care infrastructure overall.

In addition to the physical health consequences of COVID-19 infection, mental health consequences of the pandemic have been widespread. Stress, fear, and anxiety about novel contagious disease outbreaks, like COVID-19, can be immense among higher-risk groups, including HCWs and other frontline workers [16]. Being exposed to COVID-19 cases in hospitals while working, being quarantined and isolated, the death or illness of a relative or friend from COVID-19, and heightened self-perception of danger due to the lethality of the virus can all negatively impact the well-being and HRQOL of HCWs [4]. More research is needed on the best ways to support the physical and mental well-being of HCWs, particularly during a pandemic.

### Study Purpose

Our research team recently developed the Roadmap 2.0 mobile health app, which includes positive psychology-based activities for users with the goal of enhancing well-being and HRQOL for family caregivers of patients undergoing hematopoietic cell transplantation (HCT) [17-19]. The mobile randomized trial in HCT caregivers is currently ongoing (ClinicalTrials.gov NCT04094844) [20]. The Roadmap 2.0 app is configured with the Fitbit application programming interface (API) [21], which enables the collection of continuous physiological data from a Fitbit watch. In addition to the Fitbit, our research team has been using a Food and Drug Administration–approved axillary temperature wearable sensor (TempTraq, BlueSpark Technologies Inc) for monitoring patients who are high risk for complications (eg, fever or cytokine release syndrome) during HCT and chimeric antigen receptor T-cell therapy (ClinicalTrials.gov NCT04051216). As the COVID-19 pandemic rapidly disrupted health care in the United States and worldwide, our research team adapted our technology, the Roadmap 2.0 app, to be used in the HCT and cellular therapy settings for the HCW population.

Herein, we provide a detailed description of the design for a longitudinal study to test the uptake and sustained use of the Roadmap 2.0 app with positive psychology–based activities over a 30-day period in HCWs (ClinicalTrials.gov NCT04756869). We postulated that simple and intentional pleasant activities combined with daily mood and symptom reporting and use of wearable sensors could be incorporated into routine HCW practices during the COVID-19 pandemic.

## Methods

### Study Design

#### Human Participants Approval

The first diagnosed COVID-19 case at Michigan Medicine—a large, tertiary academic health system in the Midwest—was on March 10, 2020. By March 13, 2020, Michigan Medicine modified its employment work schedules where only essential, frontline HCWs were allowed into its facilities. This study was approved by the Institutional Review Board (IRB) of Michigan Medicine (IRBMED HUM00180076) on April 20, 2020, and registered on ClinicalTrials.gov (NCT04756869). At the time, all clinical research studies underwent an initial review process by the University of Michigan Office of Research Committee.

#### Overview

**Figure 1:**
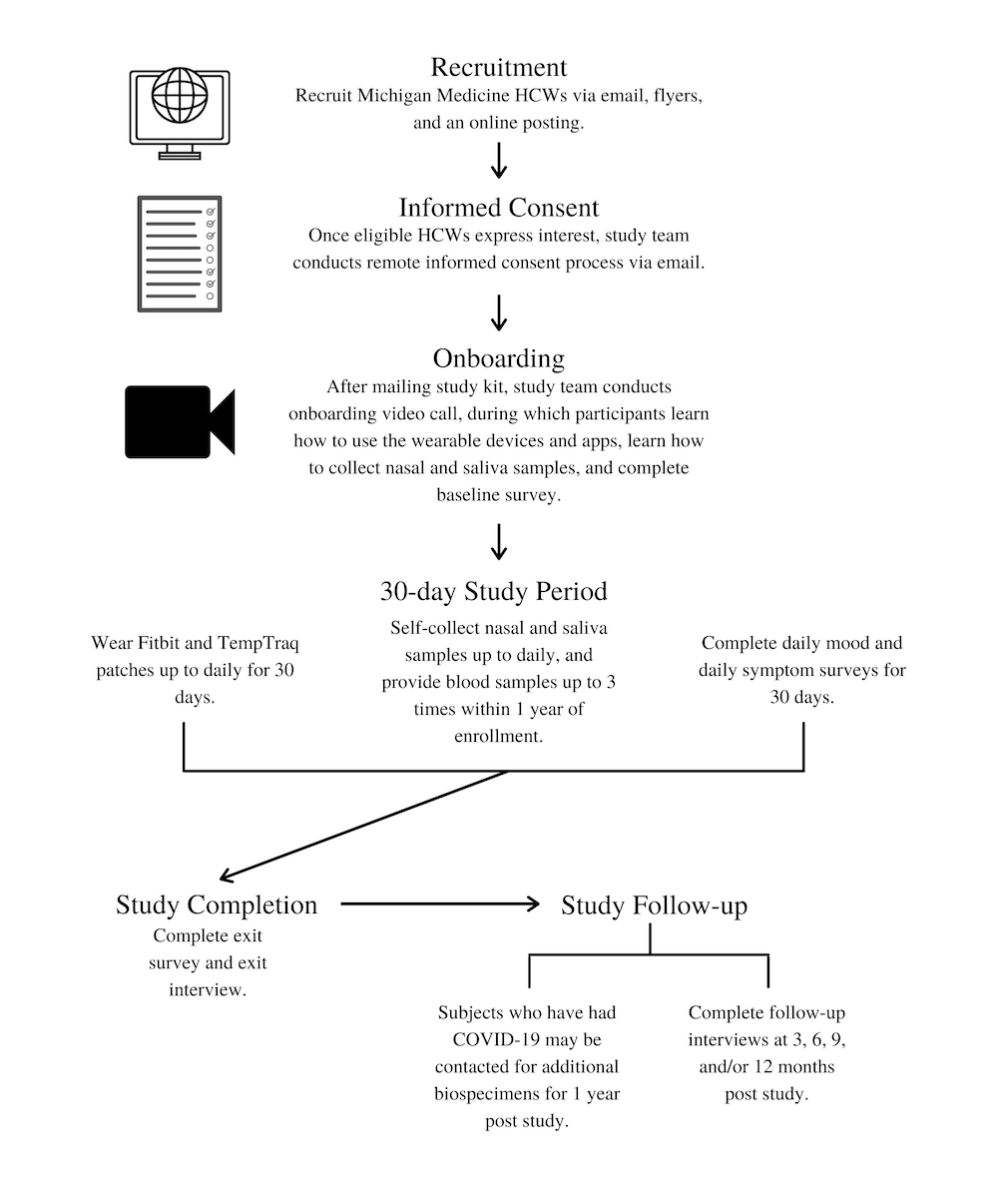
Study schematic. HCW: health care worker.

This was a prospective study of HCWs at risk for COVID-19 at a single academic institution, Michigan Medicine. In this study, participants consented to wearing a Fitbit Charge 3 smartwatch and TempTraq temperature patches continuously for up to 30 days. They could also opt in to providing nasal swabs and saliva samples daily throughout the study period and blood samples up to 3 times throughout the year after study enrollment. Finally, they completed several surveys on the smartphone-based Roadmap 2.0 app; these surveys included a baseline survey, exit survey, daily mood surveys, and daily symptom surveys. After the 30-day study period, participants were asked to participate in a semistructured qualitative exit interview. Follow-up interviews are being conducted at 3, 6, 9, and 12 months after study completion (see Figure 1 for a schematic outline of the study procedures).

#### Objectives

The primary objective was to test the feasibility of using wearable devices in HCWs. Feasibility was defined as wearing the Fitbit Charge 3 at least 8 hours per day for at least 5 days per week (~40 hours/week or 160 hours/30 days) and wearing the TempTraq patch at least 8 hours per day for at least 5 days per week (~40 hours/week or 160 hours/30 days).

The secondary objective was to assess survey completion rate, estimating that at least 50% of participants would complete the baseline survey and exit survey, and at least 50% of participants would complete at least 50% of the daily symptom surveys.

The exploratory objective was to analyze continuous heart rate and temperature data from wearable devices alongside nasal swabs, saliva, and blood samples in HCWs to facilitate the eventual development of an early prediction and detection model for COVID-19 infections.

### Participant Enrollment

#### Eligibility Criteria

Participants were required to be HCWs at Michigan Medicine who were aged at least 18 years at the time of enrollment. Additionally, they must have provided direct in-person patient care, or they must have worked in units where COVID-19 patient care occurred or was likely to occur (eg, medical assistants and custodial staff). The only exclusion criteria were unwilling or unable to comply with the study procedures or allow the study team access to health data.

#### Recruitment

The primary tools for recruitment were flyers, emails, and an online posting at UMHealthResearch.org. The study team distributed an IRB-approved recruitment flyer describing the study and providing the study team contact information. Emails containing this flyer were sent to relevant list servers, including House Officers (eg, residents and fellows). Finally, the team created an online posting at UMHealthResearch.org, through which many participants expressed interest in the study and communicated with the study coordinators.

#### Informed Consent Process

Due to COVID-19 restrictions at Michigan Medicine and other public health guidelines, informed consent was obtained remotely. Interested participants that contacted the study team received additional study information, including an IRB-approved electronic copy of the informed consent document via email. The study coordinators then provided real-time discussion of the consent via videoconference (eg, Zoom) or phone. After that discussion, the participant could sign the informed consent document and return it to the study team electronically via email.

#### Enrollment

Upon receipt of the signed informed consent document, the study coordinators assembled a study kit for the participant, including a Fitbit watch, TempTraq temperature patches, saliva kit, and nasal swab kit. They also included written instructions and shipping materials for collecting and returning the biospecimens. Finally, the study coordinators mailed study kits to participants via United Parcel Service.

After mailing study kits, study coordinators scheduled an *onboarding* video call with each participant. During the onboarding call, the study coordinators helped the participant download and log in to the study apps: Fitbit app, Roadmap 2.0 app, and TempTraq Patient app. Study coordinators reviewed how to use, charge, and sync the Fitbit watch, and how to use and place the TempTraq patches for accurate temperature readings. Study coordinators also reviewed nasal swab and saliva collection components. Finally, participants completed the initial 40-item baseline survey on their smartphones through the Roadmap 2.0 app during the onboarding video call.

### Biospecimens

#### Nasal Swabs and Saliva Collections

The study team collected only one nasal and saliva sample from each participant. These samples were self-collected by the participants at home, using collection kits provided by the study team. We used Zymo DNA/RNA Shield Nasal Swab Collection Kits and Spectrum DNA Saliva Collection Kits [22,23]. Both of these kits have a preservative that not only preserves the RNA present in the sample but also inactivates any virus that may be present in the sample.

During the onboarding video call, study coordinators instructed participants on how to collect their nasal and saliva samples. For the nasal swab, coordinators instructed participants to insert the swab into each nostril until the tip was no longer visible. Participants were then instructed to twist the swab back and forth in each nostril. They were then to place the swab into a tube that contained a reagent to preserve the sample. For the saliva sample, coordinators first instructed the participant not to eat or drink for at least 30 minutes before collecting the sample. To collect the sample, participants were instructed to spit into the collection tube up to the fill line. They were then to pour a preservative into the collection tube and mix the solutions. Finally, participants were instructed to ship their samples to the Tewari Lab at Michigan Medicine within 24 hours of collecting their samples, using the provided biospecimen packaging and shipping supplies.

#### Blood Collections

Blood collections were an optional component of this study, and participants indicated their willingness to participate in during the consent. Blood draws could occur up to 3 times over the course of 1 year following study enrollment at any Michigan blood draw station in accordance with COVID-19 guidelines.

The primary goal of collecting blood samples was to determine antibody titers to SARS-CoV-2. The team was also interested in obtaining specimens for the measurement of other immunologic analytes (eg, immune cell profiles) and to do new biomarker discovery. We proposed to collect up to 50 mL of blood in ethylenediaminetetraacetic acid tubes, serum collection tubes, or in some cases tubes with specifically targeted preservatives (eg, Cell Preparation Tubes or Streck DNA or RNA tubes). Tubes would be stored and transported at room temperature for processing within the Tewari Lab.

#### Specimen Handling, Processing, and Storage

Because we did not know the COVID-19 status of participants in real time, we developed a standard operating procedure to ensure the safety of all lab members. In summary, lab staff wore personal protective equipment at all times while handling specimens (face mask, safety glasses, lab coat, and disposable gloves), and all work handling the sample’s primary container was conducted in a biosafety cabinet. Lab surfaces were sprayed down with 70% ethanol, and UV light was turned on in the biosafety cabinet at the beginning of each day. For each sample received, the outside packaging was sprayed with 70% ethanol, and then the sample’s primary container was sprayed with 70% ethanol as well. Decontaminated samples were labeled with a study ID and stored at –80 °C. At the end of each day, lab surfaces were again sprayed with ethanol, and UV light was turned on in the biosafety cabinet once more.

### Wearable Devices

#### TempTraq Single Use Thermometer

TempTraq is a single use adhesive thermometer that continuously records axillary temperature data for 24 hours [24]. TempTraq has been tested to the American Society for Testing and Materials E1112-00 standard, which is required for all clinical digital thermometers. The temperature patch broadcasts continuous temperature data via Bluetooth 4.0 to the TempTraq mobile app. The current temperature is broadcast every 10 seconds, and the complete history of temperature data stored on the patch is broadcast every 2 minutes.

For this study, participants were asked to wear the patches continuously for up to 30 days. During the onboarding call, they were instructed on how to download and log in to the TempTraq Patient app, as well as how to properly apply the patches for accurate readings. Participants could view their temperature data in real time on their smartphone via the TempTraq Patient app. The complete TempTraq data is stored on the TempTraq Connect server for access by the study team using the TempTraq Clinician app.

#### Fitbit Charge 3

The Fitbit Charge 3 is a smartwatch fitness tracker that monitors various fitness metrics including steps, heart rate, and sleep. The Charge 3 device wirelessly connects to the patient’s smartphone via Bluetooth Low Energy and uploads data to the Fitbit app every 15 minutes [25].

For this study, participants were asked to wear the Fitbit at least 40 hours per week for 30 days. During the onboarding call, they were instructed on how to download and log in to the Fitbit app. They were also instructed to connect their Fitbit to the Roadmap 2.0 app, which was used as an interface for the study team to access the participants’ Fitbit data. The study team has access to the complete Fitbit data and can download it for analysis.

### Surveys and Interviews

#### Surveys

All surveys were Qualtrics-based and were stored on Health Insurance Portability and Accountability Act (HIPAA)–compliant U-M secure servers through the Roadmap 2.0 app. Participants were not incentivized to complete the surveys; the only incentive for study participation was keeping the Fitbit watch. Certain survey items were only conditionally displayed based on responses to other items to reduce the number of the questions. Participants were not able to review or change their survey responses. Data collection from surveys is complete as of January 2021, and data analyses are ongoing.

During the onboarding video call, participants were provided with a unique access code that was required to enter the Roadmap 2.0 app. After entering the code, participants completed a 49-item baseline survey, distributed over five screens, that automatically was pushed to the Roadmap 2.0 app on their phones. Completion of the baseline survey was required to gain access to the rest of the Roadmap 2.0 app.

For 30 days following study onboarding, study participants received daily surveys through the Roadmap 2.0 app, including a 9-item daily symptom survey and a single-item mood questionnaire. Finally, at the end of the 30-day study period, participants received and completed an 8-item exit survey through the Roadmap 2.0 app. Participants were instructed and reminded to complete these follow-up surveys, but unlike the baseline survey, they were not required to use the rest of the Roadmap 2.0 app (see Multimedia Appendix 1 for a list of all questionnaire items and interview guides).

#### Exit Interview

Participants were asked to participate in a semistructured qualitative exit interview lasting about 10-20 minutes. The interviews were conducted via videoconference or phone, and permission was asked to audio record the interviews. The exit interview included questions about the HCWs’ experiences with each of the study components and their experiences surrounding the COVID-19 pandemic.

#### Follow-up Interviews

Participants may be contacted by study coordinators to complete short follow-up semistructured interviews at the 3-, 6-, 9-, and 12-month time points after their 30-day study period was complete. These interviews included questions surrounding the participants’ overall health and well-being; their experience of the study; and their beliefs and perceptions surrounding the COVID-19 pandemic, testing, and vaccination.

#### Roadmap 2.0 App

Study participants responded to all surveys on their smartphones through the Roadmap 2.0 app, which was also used to interface with the Fitbit data through an API [21]. This app was originally developed by author SWC and colleagues as part of a project funded by a National Heart, Lung, and Blood Institute (NHLBI) R01 grant, R01HL146354-01, and is currently being evaluated in a study of caregivers of HCT patients [17-19]. As an added potential benefit to the participants, the Roadmap 2.0 app included a set of positive psychology–based activities that participants could use if they desired, including positive piggy bank, gratitude diary, savoring, pleasant activity scheduling, random acts of kindness, signature strengths, love letter, and engaging with beauty. The app also had a chat forum where participants could anonymously post and comment about themes related to the positive psychology–based activities.

### Study Completion

At the end of the initial 30-day study period, participants were asked to complete an exit survey and exit interview, in addition to follow-up interviews at 3, 6, 9, and 12 months post study. Participants were allowed to keep their Fitbits for personal use after study completion. Any Fitbit data that continues to be collected will be accessible by the study team for up to 1 year after study completion.

We proposed to collect additional nasal swabs and blood samples up to 1 year after study completion on any participants who are clinically diagnosed with COVID-19 while in the study. Participants could opt in or out on the informed consent document to allow for recontact by the study team.

### Data Collection and Analysis

#### Data Storage and Security

Participant data were stored on the wearable sensor devices and transmitted to the relevant device’s app on the participant’s smartphone via Bluetooth or Wi-Fi. Participants and their data are deidentified using coded identifiers within the devices and apps. The study team maintained deidentified participant data on a HIPAA-compliant, password-protected drive on secure university encrypted servers maintained by the Health and Information Technology Services at Michigan Medicine to protect the confidentiality of the participants.

#### Data Collection and Sharing

In addition to the study data generated from the wearable devices and survey responses, participants provided consent to access a database of COVID-19 testing results maintained by Occupational Health Services at Michigan Medicine. This access would allow the study team to determine which of the participants developed COVID-19 illness or other respiratory infections, as well as antibody titer information if it becomes available in the future. The study team could also access participants’ work schedules through hospital administrative data. Finally, the study team could access participants’ electronic medical records during the study and up to 2 years after study completion to obtain additional clinical data to correlate with the wearable sensor data and symptom reporting data.

Participants’ deidentified data and biospecimens could be shared with other researchers at the University of Michigan, around the world, and with companies. Deidentified participant data could also be used for future research studies without additional informed consent.

#### Data Analysis

Participant demographics, baseline characteristics, and daily symptom surveys will be summarized for all participants. Participant characteristics to be examined include age, gender, race, ethnicity, occupation, comorbidities, COVID-19 history, COVID-19 beliefs, and overall health and well-being.

Using computational techniques, we plan to assess the relationship between self-reported symptom data, wearable sensor data, and clinical diagnoses of respiratory illnesses, which may be COVID-19 or other types of infections. We will build on analytic approaches already developed in our other studies among oncology patients, including HCT patients who develop fevers and cell therapy patients who develop cytokine release syndrome.

We will take a multitiered approach to data analysis. This will involve initial quality control and data cleaning, data visualization, and descriptive statistics. Subsequent analyses will seek to calculate measures of correlation between the data themselves—temperature, heart rate, and symptoms data—and with clinical outcomes, particularly COVID-19 status. This aim is exploratory; we expect to obtain pilot data to power a larger subsequent study to test correlations between wearable sensor data, symptoms data, and clinical outcomes. If sufficient data are available, we may also undertake a machine learning–based analysis, such as the one we recently described for the analysis of continuous temperature data for graft-versus-host disease prediction in an animal model [26,27].

## Results

This protocol was approved by the IRB of Michigan Medicine on April 24, 2020. The first participant was enrolled on April 28, 2020, and the last participant was enrolled on December 7, 2020. We enrolled 226 HCWs within that time period. All participants have now completed the 30-day study procedures and will remain on follow-up for 2 years after completing the study period. Data collection is ongoing. Analysis of demographic and baseline characteristics has begun, and the rest of the analysis is ongoing.

The COVID-19 pandemic affected the execution of this study in a number of ways. All recruitment, consenting, onboarding, and participant follow-up were conducted remotely via videoconference, phone, and email. Although we proposed to collect saliva and nasal samples daily and blood samples up to three times throughout the study, due to COVID-19 restrictions at the University of Michigan in conjunction with limited resources, we only collected one saliva sample, one nasal sample, and no blood samples from our participants. Finally, due to the ever-changing nature of the pandemic, the study was carried out in two segments. An initial cohort of 20 HCWs was enrolled in April and May 2020 and participated in the wearable device portion of the study, in addition to surveys and interviews. Informed consent for biospecimen collection was later added to the study, after which a second larger cohort of HCWs was enrolled between August and December 2020.

## Discussion

In this study, HCWs wore a smartwatch and temperature patches, completed daily symptom surveys, and submitted biospecimens for analysis, with the aim of assisting HCWs in self-monitoring for COVID-19 infection. We enrolled 226 HCWs between April 28 and December 7, 2020. All participants have completed the 30-day study procedures, with the last participant reaching day 30 on January 16, 2021. Data collection will continue through January 2023. Data processing and analyses are ongoing at the time of writing this manuscript.

Studying the feasibility of wearable sensor use and daily smartphone-based symptom logging is timely, as the use of digital health technologies has surged during the COVID-19 pandemic [28,29]. One year since the start of the pandemic, the majority of HCWs have adjusted to social distancing and remote working and learning. A return to some sense of normalcy in the near future is anticipated with wide scale vaccine distribution efforts. However, it remains uncertain what the new normal may entail. It is imperative that the wellness and HRQOL of HCWs, their families, and their colleagues continue to be prioritized as health care systems navigate through the broad, sweeping changes that the pandemic has brought worldwide. Thus, the role of wearables and mobile health apps in health care is likely to continue growing in the coming years.

The COVID-19 pandemic has affected health care systems in many ways. In particular, the health care workforce has been impacted by the relatively high incidence of COVID-19 among HCWs [10]. There have been wide-ranging physical and mental health implications for all HCWs working during the COVID-19 pandemic, including what is now described as the long-haul syndrome [30]. Psychological distress, fear, and burnout related to working during the COVID-19 pandemic are common among HCWs [31-34]. As such, many recent mobile health studies have targeted the mental health of HCWs during COVID-19—from telepsychiatry to mindfulness apps [35-38]. Our study builds upon this literature with the positive psychology–based activities included in the Roadmap 2.0 app. Additionally, the literature surrounding the use of wearable sensors to predict or detect COVID-19 is growing. Several studies have demonstrated that wearable sensor data and symptoms data may be useful for the early detection of COVID-19 [39-42]. However, research is limited on the utility of this kind of data for monitoring HCWs for infection.

At first glance, the movement toward digital health technologies seems apt to increase access to health care by increasing the ease and accessibility of consulting with a physician or monitoring one’s health. Indeed, virtual visits tend to be more flexible in terms of time and location than in-person consultations. However, telemedicine and other forms of digital health technologies require internet access and knowledge of how to use the digital health platform. Research shows that lack of internet access and digital illiteracy are both correlated with low income, being a racial or ethnic minority, being older than 65 years, or speaking a primary language other than English [43]. Thus, the equitable distribution of digital health solutions is limited by digital access and digital literacy. Furthermore, just as patients vary in this parameter, HCWs have varying levels of digital literacy. For this reason, mobile health interventions in both patients and HCWs need to consider these factors.

This study has several limitations. First, we did not address the digital divide. HCWs are a relatively highly educated group, which correlates with higher digital literacy. For this reason, the protocol may not be easily adapted to other populations outside of HCWs. Second, our study is subject to selection bias by which technologically savvy HCWs may have been more likely to enroll than those with lower digital literacy. Thus, results surrounding the feasibility of using the wearable sensors, which requires some technological expertise, may be positively skewed. Finally, this was a study of HCWs at a single institution, Michigan Medicine, which means unique institutional factors may influence our results. Future studies should focus on strategies to mitigate the digital divide and expand the reach of mobile health interventions.

This protocol will reveal key data on the feasibility of using wearable sensors and symptom reporting among HCWs. These data are important for evaluating the viability of this kind of intervention for monitoring HCWs for infection in the real world. Additionally, we hope that it will add valuable pilot data to the growing literature surrounding wearable sensor and symptoms data for the early detection of COVID-19 infection. With its unique combination of wearables data, symptoms data, and biospecimens, we anticipate that this study will illuminate effective HCW monitoring practices, which may be useful for future pandemic preparedness.

## Appendix

Multimedia Appendix 1 Survey measures.

## Abbreviations

API: application programming interface
HCT: hematopoietic cell transplantation
HCW: health care worker
HIPAA: Health Insurance Portability and Accountability Act
HRQOL: health-related quality of life
IRB: Institutional Review Board
NHLBI: National Heart, Lung, and Blood Institute
