# Supplementary material for: Monitoring Health Care Workers at Risk for COVID-19 Using Wearable Sensors and Smartphone Technology: Protocol for an Observational mHealth Study
Source: JMIR Res Protoc. 2021 May 12;10(5):e29562. doi: 10.2196/29562 (PMC8117956; doi:10.2196/29562)
Supplement: Multimedia Appendix 1 [file resprot_v10i5e29562_app1.docx]

Baseline Survey

1. What is your age?: _______
2. What is your gender
   1. Female
   2. Male
   3. Not Listed (Please specify ___________)
3. Are you of Spanish, Hispanic, or Latino origin or descent?
   1. Yes
   2. No
4. What is your race? (choose all that apply)
   1. White
   2. Black or African American
   3. Asian
   4. Native Hawaiian or other Pacific Islander
   5. American Indian or Alaska Native
   6. Other (please specify) __________
5. What is your marital status?
   1. Single, never married
   2. Married or in domestic partnership
   3. Widowed
   4. Divorced
   5. Separated
6. What is the highest grade or level of schooling that you have completed?
   1. Some high school, did not graduate
   2. High school graduate or GED
   3. Some college or 2-year degree
   4. 4-year college graduate
   5. More than 4-year college degree
   6. Not applicable
7. Including yourself, how many people live in your household:_______
8. What is the makeup of the people living in your household (check all that apply and include number)
   1. Children: __
   2. Spouse: __
   3. Other family member(s): ____
   4. Roommates (not family):____
   5. I live alone
9. What is your current occupation?
   1. Physician
   2. Physicians Assistant
   3. Nurse Practitioner
   4. Nurse
   5. Pharmacist
   6. Phlebotomist
   7. Clinical Fellow
   8. Resident
   9. Medical Technician
   10. Housekeeping Staff Member
   11. If not listed above please specify:__________
10. What is your current employment status?
    1. Employed full-time (40 or more hours per week)
    2. Employed part time (up to 39 hours per week)
11. Have you been diagnosed with COVID19?
    1. Yes
    2. No
12. If yes: What was the date of diagnosis? ___________
13. If yes: Did you require hospitalization for COVID19?
    1. Yes
    2. No
14. If you required hospitalization, how long were you hospitalized? (Please indicate if hospitalization occurred more than once) _______________
15. Please select the symptoms that you experienced while having COVID19.
    1. Fever
    2. Chills
    3. Shortness of breath
    4. New or worsening cough
    5. Sore throat
    6. Body aches
    7. Vomiting
    8. Diarrhea
    9. Loss of smell
    10. Loss of taste
    11. None of the above
    12. Other
16. How would you rate your overall health during the past year?

Very Poor Excellent

1 2 3 4 5 6 7

1. How would you rate your overall quality of life during the past year?

Very Poor Excellent

1 2 3 4 5 6 7

1. Do you have any of the following health conditions? (please check all that apply)
   1. Asthma
   2. COPD
   3. Obstructive sleep apnea
   4. Pulmonary hypertension
   5. Other lung disease (please specify): _________
   6. Diabetes
   7. Heart disease
   8. High blood pressure
   9. High cholesterol
   10. Kidney disease
   11. Cancer
   12. Hepatitis
   13. Fatty liver disease
   14. Crohn’s disease or ulcerative colitis
   15. Multiple sclerosis (MS)
   16. Lupus or other autoimmune condition
   17. Stroke
   18. Arthritis
   19. Migraine headaches
   20. Seasonal allergies
   21. Insomnia or other sleep disorder
   22. Anxiety
   23. Depression
   24. Post-traumatic stress disorder
   25. Other mental health disorder
   26. Brain injury
   27. Other (please specify) __________
   28. None of the above
2. Are you currently living with someone who has tested positive for COVID19?
   1. Yes
   2. No
3. Do you live with someone who falls into the high-risk category for COVID19 (e.g., elderly, immunocompromised, multiple health problems)?
   1. Yes
   2. No
4. How many prescription medications do you take?
   1. None
   2. 1-2
   3. 3-5
   4. 6 or more
5. Do you currently smoke cigarettes?
   1. Yes
   2. No
6. Number of years smoking cigarettes:
   1. ______
   2. I do not smoke cigarettes
7. Average number of pack(s) smoked per day:
   1. __________Pack(s) of cigarettes
   2. I do not smoke cigarettes

Within the past year how often have you….

1. Used Marijuana
   1. Did not use
   2. Once/Year
   3. 6 times/Year
   4. Once/Month
   5. Twice/Month
   6. Once/Week
   7. Three times/Week
   8. Every Day
2. Engaged in vaping (Juul, e-cigarettes, weed pens, etc.)
   1. Did not use
   2. Once/Year
   3. 6 times/Year
   4. Once/Month
   5. Twice/Month
   6. Once/Week
   7. Three times/Week
   8. Every Day
3. Consumed alcohol
   1. Did not use
   2. Once/Year
   3. 6 times/Year
   4. Once/Month
   5. Twice/Month
   6. Once/Week
   7. Three times/Week
   8. Every Day
4. In the last 12 months how often have you participated in some form of active physical exercise (e.g., running, yoga, pilates, cross-fit, dance)?
   1. Daily
   2. 3 to 4 times per week
   3. 1 to 2 times per week
   4. 1 to 2 times per month
   5. Not at all

Work Schedule/COVID

1. Where did you work, or are you scheduled to work, Today?
   1. Inpatient Unit
      1. Which unit?: ________
   2. Outpatient Unit
      1. Which unit or clinic?: ________
   3. I did not work at the hospital or clinic today
2. Which shift did you work, or are you scheduled to work, Today?
   1. Day shift
   2. Night shift
   3. Evening shift
   4. I did not work at the hospital or clinic today
3. Are you assigned in the next 4 weeks to work on a unit or in an area with patients who have tested positive or are presumed to be positive for COVID19?
   1. Yes
   2. No
4. Are you currently having face-to-face contact with patients who have tested positive for or are presumed to be positive for COVID19?
   1. Yes
   2. No
5. Are you experiencing any of the following symptoms (check all that apply):
   1. Fever
   2. Chills
   3. Shortness of breath
   4. New or worsening cough
   5. Sore throat
   6. Body aches
   7. Vomiting
   8. Diarrhea
   9. Loss of smell
   10. Loss of taste
   11. None of the above
   12. Other
6. Have you been tested for COVID19?
   1. Yes
   2. No
7. What type of testing have you had done? (if yes to question above)
   1. Swab for PCR detection
   2. Serology/antibody
   3. Other
8. How many times have you been tested for COVID19 with a swab for PCR detection? _______
9. What was the result to the serology test? (if yes to serology)
   1. Positive
   2. Negative
   3. Test results are still pending
10. What was the result of the PCR detection? (if yes to swab for PCR detection) (for each test)
    1. Positive
    2. Negative
    3. Test results are still pending
11. When was the testing for COVID19? (if yes to nasal swab testing for each test)
    1. 1 week ago
    2. Between 1-2 weeks ago
    3. Between 2-4 weeks ago
    4. Between 1-3 months ago
    5. Greater than 6 months ago

Questions related to the experience and value from participating in the study; questions related to beliefs and expectations.

1. I feel stressed about the COVID-19 pandemic.
   1. Strongly agree (1)
   2. Somewhat agree (2)
   3. Neither agree nor disagree (3)
   4. Somewhat disagree (4)
   5. Strongly disagree (5)
2. I feel optimistic about the future
   1. Strongly agree (1)
   2. Somewhat agree (2)
   3. Neither agree nor disagree (3)
   4. Somewhat disagree (4)
   5. Strongly disagree (5)
3. I feel I am likely to become infected with the novel coronavirus.
   1. Strongly agree (1)
   2. Somewhat agree (2)
   3. Neither agree nor disagree (3)
   4. Somewhat disagree (4)
   5. Strongly disagree (5)
   6. I have already had a positive COVID-19 test
4. I worry about my own safety if I were to become infected with the novel coronavirus.
   1. Strongly agree (1)
   2. Somewhat agree (2)
   3. Neither agree nor disagree (3)
   4. Somewhat disagree (4)
   5. Strongly disagree (5)
   6. Not Applicable because I have already had a positive COVID-19 test
5. I worry about the safety of patients if I were to become infected with the novel coronavirus.
   1. Strongly agree (1)
   2. Somewhat agree (2)
   3. Neither agree nor disagree (3)
   4. Somewhat disagree (4)
   5. Strongly disagree (5)
6. I worry about my family's safety if I were to become infected with the novel coronavirus
   1. Strongly agree (1)
   2. Somewhat agree (2)
   3. Neither agree nor disagree (3)
   4. Somewhat disagree (4)
   5. Strongly disagree (5)
   6. Not Applicable because I have already had a positive COVID-19 test
7. Approximately how many times per day have you checked the news on average during the last 2 weeks?
   1. 0 -1 (1)
   2. 2 - 4 (2)
   3. 5 - 7 (3)
   4. > 7 (4)
8. What is your primary type of cell phone?
   1. Apple iPhone
   2. Android smartphone (e.g., Samsung, Google)
   3. Microsoft windows smartphone (e.g., Microsoft, HP Elite)
   4. Cell phone that is not a smartphone
   5. Other (please specify) __________
   6. I do not own a cell phone
9. Do you own a fitness or smart watch? (check all that apply)
   1. Apple iWatch
   2. Fitbit Fitness Watch
   3. Garmin Fitness Watch
   4. Other (please specify) _________
   5. I do not own a fitness or smart watch
10. Which of the following kinds of health or wellness-related apps do you use on your smartphone or tablet? (please check all that apply)
    1. Fitness workouts
    2. Counting steps
    3. Nutrition (e.g., tracking calories, recording diet)
    4. Meditation or stress management
    5. Sleep
    6. Other (please specify) __________

Daily Survey: Symptom Tracking (Day 1 → Day 30)

1. Where did you work, or are you scheduled to work, Today?
   1. Inpatient Unit
      1. Which unit?: ________
   2. Outpatient Unit
      1. Which unit or clinic?: ________
   3. I did not work at the hospital or clinic today
2. Which shift did you work, or are you scheduled to work, Today?
   1. Day shift
   2. Night shift
   3. Evening shift
   4. I did not work at the hospital or clinic today
3. Are you assigned in the next 4 weeks to work on a unit or in an area with patients who have tested positive for or are presumed to have COVID19?
   1. Yes
   2. No
4. Are you currently having face-to-face contact with patients who have tested positive for or are presumed to have COVID19?
   1. Yes
   2. No
5. Are you experiencing any of the following symptoms (check all that apply):
   1. Fever
   2. Chills
   3. Shortness of breath
   4. New or worsening cough
   5. Sore throat
   6. Body aches
   7. Vomiting
   8. Diarrhea
   9. Loss of smell
   10. Loss of taste
   11. None of the above
   12. Other
6. Have you been tested for COVID19 since the last daily survey you have taken (including serology)?
   1. Yes
   2. No
7. What type of testing have you done? (if yes to being tested)
   1. Swab for PCR detection
   2. Serology/ antibody
   3. Other
8. What was the result of the serology testing? (if yes to serology)
   1. Positive
   2. Negative
   3. Test results are still Pending
9. What was the result of the PCR testing? (if yes to PCR testing)
   1. Positive
   2. Negative
   3. Test results are still Pending

Exit Survey

1. I feel stressed about the COVID-19 pandemic.
   1. Strongly agree (1)
   2. Somewhat agree (2)
   3. Neither agree nor disagree (3)
   4. Somewhat disagree (4)
   5. Strongly disagree (5)
2. I feel optimistic about the future
   1. Strongly agree (1)
   2. Somewhat agree (2)
   3. Neither agree nor disagree (3)
   4. Somewhat disagree (4)
   5. Strongly disagree (5)
3. I feel I am likely to become infected with the novel coronavirus.
   1. Strongly agree (1)
   2. Somewhat agree (2)
   3. Neither agree nor disagree (3)
   4. Somewhat disagree (4)
   5. Strongly disagree (5)
   6. I have already had a positive COVID-19 test
4. I worry about my own safety if I were to become infected with the novel coronavirus.
   1. Strongly agree (1)
   2. Somewhat agree (2)
   3. Neither agree nor disagree (3)
   4. Somewhat disagree (4)
   5. Strongly disagree (5)
   6. Not Applicable because I have already had a positive COVID-19 test
5. I worry about safety of patients if I were to become infected with the novel coronavirus.
   1. Strongly agree (1)
   2. Somewhat agree (2)
   3. Neither agree nor disagree (3)
   4. Somewhat disagree (4)
   5. Strongly disagree (5)
6. I worry about my family's safety if I were to become infected with the novel coronavirus
   1. Strongly agree (1)
   2. Somewhat agree (2)
   3. Neither agree nor disagree (3)
   4. Somewhat disagree (4)
   5. Strongly disagree (5)
   6. Not Applicable because I have already had a positive COVID-19 test
7. Approximately how many times per day have you checked the news on average during the last 2 weeks?
   1. 0 -1 (1)
   2. 2 - 4 (2)
   3. 5 - 7 (3)
   4. > 7 (4)
8. Have you personally experienced disadvantages, benefits, or both as a result of the COVID-19 pandemic?
   1. Disadvantages, no benefits
   2. More disadvantages, but there have been some advantages
   3. Roughly equal disadvantages and benefits
   4. More benefits, but there have been some disadvantages
   5. Only benefits, no disadvantages

Exit Interview: Audio-Recorded, Semi-Structured Qualitative Interview

Participant Experience:

In your opinion…..

1. What worked with our study?
2. What did not work in the study?
3. Which aspects of the study do you really like or appreciate?
4. What are ways in which we could improve our study?

Study Value/Adherence:

1. Were there any unexpected values or benefits you got from the study?
2. How successful was the virtual onboarding to the study?
3. How was your experience with the Roadmap app?
4. How was your experience with the TempTraq app?
5. How was your experience with the Fitbit?
6. How was your experience with the nasal swabs?
7. How was your experience with the saliva collections?
8. Did you find the study burdensome? If so, what aspects? In which ways would you modify the study to be less burdensome?
9. Did you find the reminders useful or were there too many reminders?

Thoughts about COVID:

1. In what ways has COVID-19 negatively affected you personally, if any?
2. In what ways has COVID-19 positively affected you personally, if any?
3. What, if any, coping mechanisms did you use to handle COVID-19?
4. Do you personally know anyone who is infected with COVID-19? How did that affect you?
5. Were you ever redeployed to another unit due to COVID-19? How did that affect you?
6. What practices did you use to monitor your health and keep you and your family safe?
7. Any additional thoughts that you would like to add?

Follow-Up Interviews (3, 6, 9, 12-month time points): Semi-Structured Phone Interview

Overall Health and Wellbeing:

1. Have you had any significant changes in your health since you last spoke with the study team?
2. Have you been diagnosed with COVID-19 since the last time we spoke with you?
   1. If so, when (approximate date)?
   2. What symptoms did you initially experience prior to your diagnosis that prompted testing?
   3. Were you hospitalized? If so, how long was your admission (approximate dates)?
3. How would you rate your overall wellbeing since you last spoke with the study team (positive or negative emotions related to COVID pandemic, feelings of burnout etc..)?

Perceptions of COVID Testing and Monitoring:

1. Do you feel there is adequate COVID testing available?
2. What are your thoughts about frequent at-home testing for routine monitoring?
3. Any additional thoughts about the role of wearable devices in monitoring for COVID or ideas about COVID testing in general?
